# Mortuary Reports

**Published:** 1851-07

**Authors:** 


*Mortuary Reports.*—The Common Council of Buffalo have lately passed an ordinance requiring sextons to keep a register of burials, with the causes of deaths, and report the same regularly to the Board of Health; and by the latter body it has been resolved to publish the results monthly.

The importance of this species of statistics is apparent enough to every physician of decent intelligence, and is fully appreciated by every intelligent member of the community so soon as the subject is brought to their reflections. To render the statistics valuable, however, it is evident that the utmost attainable accuracy is indispensable. The liability to errors especially relates to the causes of death. It is difficult to render the returns in this respect perfectly reliable. A considerable share of mortality occurs among patients attended by irregular practitioners, and it cannot be claimed that all regular physicians are invariably unerring in diagnosis. Notwithstanding these difficulties, the certificate of the medical attendant affords the best, and indeed the only practicable means of determining the diseases in cases of death. Statistics made up of rumors, or the opinions of friends, on this point, would be comparatively worthless.

The regulations at present adopted in this city, it appears, do not make adequate provisions for this object, and a memorial signed as we are informed by all the regular physicians in the city, (with a single exception,) to whom it has been presented, has been addressed to the city corporation, asking that Sextons shall be required in all cases before burial to procure from the practitioner attending during the last illness, in writing, with his name attached, the name of the disease of which the patient died. This is the course pursued in other cities in which the subject has received proper attention. It is to be hoped that the City Council will comply with the requests of the Physicians, and that the regulation will be strictly enforced.*

The following is the Report of deaths and diseases for the month of May last:

*Board of Health, Buffalo, June*, 1851.—Number of deaths reported to the Board for the month of May: males 50; females 36; total 86. Age, adults 40; children 46. Nation—Americans 33; Germans 24; Irish 16; English 6; Scotch 4; French 1; Swiss 1; Norwegian 1. Diseases—apoplexy 1; bronchitis 1; consumption 19; convulsions 7; dentition 1; delirium tremens 4; disease of heart 1; dropsy 1; drowned 1; fever 7; hydrocephalus 4; inflammation of brain 1; old age 3; paralysis 1; pneumonia 2; scarlet fever 9; scrofula 1; small pox 1; still born 2; unknown 10; ulcerated throat 1; whooping cough 1.

By order of the Board,

W. L. G. SMITH, Clerk.

* Since the above was written the ordinance asked for has been passed by the Common Council.

